# A combined antibody and DNA assay for EBV infection in children

**DOI:** 10.3389/fped.2022.989193

**Published:** 2022-08-25

**Authors:** Yulu Yang, Yafei Zhu

**Affiliations:** ^1^Department of Pediatrics, The Affiliated Hospital of Hangzhou Normal University, Hangzhou, China; ^2^Department of Neonatology, Women's Hospital, Zhejiang University School of Medicine, Hangzhou, China

**Keywords:** EBV, antibody, EBV-DNA, children, influencing factors

## Abstract

**Objective:**

This paper studied the Epstein–Barr virus (EBV) infection status and influencing factors among children using a combined detection of specific antibodies and DNA.

**Methods:**

We retrospectively analyzed children who visited the Affiliated Hospital of Hangzhou Normal University from January 2019 to December 2020, and correlations between the social environment and clinical data were analyzed.

**Results:**

The cumulative positive rates of specific antibody, DNA, and combined detection of EBV were 52.4%, 39.5%, and 54.0% (*P* = 0.001), respectively. The current infection rate was 15.7%, and the peak of infection occurred in the preschool group (*P* = 0.021). After adjusting for confounding factors, the number of siblings (*OR* = 1.550) and family members who smoke (*OR* = 1.524) were independent risk factors for EBV infection, whereas parents with a higher education level *(OR* = 0.493, *OR* = 0.316), longer breastfeeding time (*OR* = 0.578) and dedicated tableware (*OR* = 0.573) were independent protective factors.

**Conclusion:**

A combination of antibody and DNA tests may be beneficial for the diagnosis of EBV infection. The EBV infection rate in children at our hospital was lower than the national average. Furthermore, the infection rate is closely related to the number of siblings, regardless of whether family members smoke, the status of parents' education, breastfeeding duration, and meal patterns. Overall, prevention measures should focus on the preschoolers.

## Introduction

The Epstein–Barr virus is a human lymphotropic herpes virus that causes a lifelong infection in approximately 95% of the global population (1). The primary EBV infection is more common in children and adolescents, and most cases are asymptomatic and atypical infections (2, 3), which are easily missed and misdiagnosed. Therefore, strengthening the monitoring of EBV in children can provide early clinical anti-infective treatment and prevention. Presently, an important method for judging EBV infection is to measure EBV-related antibodies. However, determining EBV infection by detecting EBV-DNA is gradually becoming more common in clinical practice. There is currently a lack of research on EBV infection in a large sample size of children with combined DNA and antibody detection in the Hangzhou area. This paper retrospectively analyzed the data of 1,744 children who were treated in the Affiliated Hospital of Hangzhou Normal University from January 2019 to December 2020 that were tested for EBV-specific antibodies and EBV-DNA.

## Subjects and methods

### Subjects

A total of 1,744 children aged 0–14 years who were tested for both EBV-specific antibodies and DNA participated in this study at the pediatrics department of the Affiliated Hospital of Hangzhou Normal University from January 2019 to December 2020. The subjects included 936 males and 808 females. The medical history and general information of the research subjects were recorded in detail including sex, age, siblings, family members' smoking status, breastfeeding duration, meal patterns, parents' educational levels, and housing conditions. Exclusion criteria included any antiviral drug treatment before the test, severe immunodeficiency or application of related immunosuppressants, a recent history of blood or blood product transfusion, basic diseases, such as malignant tumor, twins, premature birth; congenital malformation, and severe organ insufficiency. This study was approved by the medical ethics committee of the hospital, and all of the children's guardians signed the informed consent forms.

### EBV-specific antibody test and EBV-DNA PCR (polymerase Chain reaction) test

Venous blood samples (4 mL each) were collected from the subjects and 2 mL was placed in a vacuum blood collection tube. We used an enzyme-linked immunosorbent assay (ELISA) (EUROIMMUN, Germany) to detect EBV capsid antigen IgM antibody (EBV-CA-IgM), EBV capsid antigen IgG antibody (EBV-CA- IgG), EBV early antigen IgM antibody (EBV-EA-IgM), and nuclear antigen IgG antibody (EBV-NA-IgG) to determine the infection stage as follows: current infection, previous infection and non-infection (4). The remaining 2 mL was placed in an EDTA tube (containing anticoagulant), and 1 mL of plasma was separated. The EBV-DNA in the plasma (1 mL) was measured by real-time fluorescence quantitative PCR technology, and 1 mL was used for detecting EBV-DNA in peripheral blood mononuclear cells (PBMCs). The reagents were provided by SANSURE BIOTECH INC. The analytical instrument was a SLAN-96S of Shanghai HONGSHI Medical Tech. EBV-DNA copies > 400 copies/mL were considered positive.

### Statistical analysis

We used Excel to organize the data and to establish a reliable database and SPSS 25.0 software for statistical analysis. The rate comparison was performed using the chi-squared test. We performed univariate analysis and multivariate logistic regression analysis on the related factors that could affect EBV infection. *P* < 0.05 was used to indicate that the difference was statistically significant.

## Results

### Epidemiological characteristics of EBV infection

The cumulative infection rates of EBV-specific antibodies and DNA tests were 52.4% (914/1,744) and 39.5% (689/1,744), respectively, and the combined detection was 54.0% (941/1,744). The difference between the tests was statistically significant (*P* < 0.01). Additionally, the cumulative EBV infection rate increased with age; the EBV infection rate of 10-year-old children was 91.4%, and the cumulative infection rate of children under the age of 6 years was 47.5% (686/1,445). The current infection rate was 15.7% (275/1,744) (Table 1).

**Table 1:** Age distribution of all participants.

	**0**	**1 y**	**2 y**	**3 y**	**4 y**	**5 y**	**6 y**	**7 y**	**8 y**	**9 y**	**10 y**	**11 y**	**12 y**	**13 y**
Current infection	16	24	40	63	34	28	23	12	16	7	5	3	3	1
Previous infection	8	44	64	121	96	62	63	72	41	37	27	11	9	11
Cumulative infection	24	68	104	184	130	90	86	84	57	44	32	14	12	12
Total number of cases	152	224	263	353	211	132	110	104	65	52	35	18	13	12

The subjects were divided by age into an infant group (0– <1-year-old), a toddler group (1–<3 years old), a preschool age group (3~ < 7 years old), and a school-age group (7–13 years old). The preschool group had the highest current infection rate (*P* < 0.05). Among the 1,744 children, the infection rate was 53.8% in boys and 54.1% in girls, and there was no significant difference between the male and female infection rates (*P* > 0.05). Furthermore, there was no significant difference in the current infection rate of EBV in different seasons (*P* > 0.05) (Table 2).

**Table 2:** Comparison of the EBV infection rate by sex, age, and seasonality.

**Group**	**Number of cases**	**Number of infected cases**	**Infection rate (%)**	**x^2^**	***P-*value**
Sex					
Male	936	504	53.8	0.010	0.921
Female	808	437	54.1		
Age					
Infant period	152	24	15.8	290.510	0.000
Toddler period	487	172	35.3		
Preschool period	806	490	60.8		
School-age period	299	255	85.3		
Age*					
Infant period	152	16	10.5	9.758	0.021
Toddler period	487	64	13.1		
Preschool period	806	148	18.4		
School-age period	299	47	15.7		
Season*					
Spring	110	24	21.8	3.916	0.271
Summer	515	76	14.8		
Autumn	632	103	16.3		
Winter	487	72	14.8		

* Indicates the number of current infections.

### Influencing factors of EBV infection

#### Univariate analysis

There was no relationship between renting a house and EBV infection (*P* > 0.05). However, the number of siblings (*P* < 0.01), parents' educational level (all *P* < 0.01), the duration of breastfeeding (*P* < 0.05), smoking status of family members (*P* < 0.01), and meal patterns (*P* < 0.01) were all associated with EBV infection (Table 3).

**Table 3:** Univariate logistic regression to determine the risk of EBV infection based on participant characteristics.

**Group**	**Number of cases**	**Number of infected cases**	**Infection rate (%)**	***OR* (95% CI)**	***P-*value**
Renting					0.151
No	1,261	667	52.9	1	
Yes	483	274	56.7	1.168 (0.945~1.442)	
Number of children					0.000
1	821	395	48.1	1	
2	846	499	59.0	1.551 (1.278~1.882)	0.000
≥ 3	77	47	61.0	1.690 (1.048~2.725)	0.032
Father's education					0.000
Junior high school or below	235	154	65.3	1	
High school	290	192	66.2	1.043 (0.726~1.498)	0.819
College	275	184	66.9	1.077 (0.746~1.554)	0.694
Undergraduate	725	313	43.2	0.405 (0.298~0.549)	0.000
Master's degree and above	218	98	45.0	0.435 (0.298~0.635)	0.000
Mother's education					0.000
Junior high school or below	201	162	80.6	1	
High school	236	152	64.4	0.435 (0.281~0.676)	0.000
College	439	239	54.4	0.288 (0.193~0.428)	0.000
Undergraduate	729	323	44.3	0.192 (0.131~0.280)	0.000
Master's degree and above	139	65	46.8	0.211 (0.130~0.343)	0.000
Breastfeeding duration (months)*					0.013
Never	131	88	67.2	1	
≤ 6 m	323	181	56.0	0.623 (0.407~0.953)	0.029
> 6 m	1,240	666	53.7	0.567 (0.387~0.830)	0.004
Have a family member that smokes					0.000
No	940	459	48.8	1	
Yes	804	482	60.0	1.569 (1.297~1.898)	
Dedicated tableware					0.000
No	460	305	66.3	1	
Yes	1,284	636	49.5	0.499 (0.399~0.623)	

* 50 samples of 0–6 m were excluded from the total number of samples due to an inability to define the duration of breastfeeding in children younger than 6 months.

#### Multifactor analysis

Incorporating the above factors into a multivariate logistic regression analysis revealed that more siblings (OR = 1.550, 95% CI: 1.226–1.960) and family members smoking (OR = 1.524, 95% CI: 1.244–1.868) were EBV-infected independent risk factors. Higher parental education (OR = 0.493, 95% CI: 0.395–0.614; OR = 0.316, 95% CI: 0.216–0.462), longer breastfeeding duration (OR = 0.578, 95% CI: 0.396–0.844), and having dedicated tableware (OR = 0.573, 95% CI: 0.452–0.726) were independent protective factors for EBV infection (Table 4).

**Table 4:** Multivariate logistic regression of EBV infection risk based on participant characteristics.

**Group**	**Number of cases**	**Number of infected cases**	**Infection rate (%)**	***OR* (95% CI)**	***P-*value**
Number of children					0.001
1	821	395	48.1	1	
2	846	499	59.0	1.542 (1.215~1.957)	0.000
≥ 3	77	47	61.0	1.642 (0.971~2.774)	0.064
Renting					0.963
No	1,261	667	52.9	1	
Yes	483	274	56.7	0.994 (0.786~1.258)	
Father's education					0.000
Junior high school or below	236	154	65.3	1	
High school	290	192	66.2	0.972 (0.665~1.421)	0.883
College	275	184	66.9	1.164 (0.791~1.714)	0.440
Undergraduate	725	313	43.2	0.503 (0.362~0.698)	0.000
Master's degree or above	218	98	45.0	0.592 (0.386~0.908)	0.016
Mother's education					0.000
Junior high school and below	201	162	80.6	1	
High school	236	152	64.4	0.475 (0.301~0.750)	0.001
College	439	239	54.4	0.342 (0.226~0.517)	0.000
Undergraduate	729	323	44.3	0.255 (0.171~0.379)	0.000
Master's degree and above	139	65	46.8	0.315 (0.187~0.529)	0.000
Breastfeeding duration (months)*					0.013
Never	131	88	67.2	1	
≤ 6	323	181	56.0	0.623 (0.407~0.953)	0.029
> 6	1,240	666	53.7	0.567 (0.387~0.830)	0.004
Have a family member that smokes					0.000
No	940	459	48.8	1	
Yes	804	482	60.0	1.524 (1.244~1.868)	
Dedicated tableware					0.000
No	460	305	66.3	1	
Yes	1,284	636	49.5	0.573 (0.452~0.726)	

* 50 samples of 0–6 m were excluded from the total number of samples. Univariate logistic regression analysis was performed alone when it was clear that there was no significant relationship with other factors through the chi-square test.

## Discussion

Specific antibodies and DNA detection of EBV infection have advantages and limitations, respectively. EBV infection produces a series of specific antibodies. When combined with the detection of multiple antibodies, a multidimensional data analysis can be performed to distinguish between current infection, previous infection, and non-infection status. However, 5–10% of infected people do not produce EBV-NA-IgG antibodies (5). Young children (especially those under the age of four), immunosuppressed, or immunodeficient children may not produce specific antibodies. Also, the virus sometimes cannot stimulate the body to produce corresponding viral antibodies that reach the lower limit of detection (6).

DNA testing is direct evidence of EBV infection and compensates for the shortage of EBV-specific antibody testing (7), but DNA testing is effective only when the virus is active. Most previous studies were only conducted through a single antibody or DNA detection method, which leads to misdiagnosis. Therefore, this study examined four specific antibodies and DNA tests, and demonstrated that this combined test can comprehensively detect and monitor EBV infection.

The cumulative infection rate of EBV in children in this study was 54.0%, which was lower than the EBV infection rate (84.93%) of 3,277 Jilin children as reported by Pan (8). This result may be related to the age distribution of the enrolled patients. Another reason for this might be the development of the economy and the improvement of living standards, which may causing the EBV infection rate to gradually decrease (9). In this study, the EBV infection rate of children under the age of six was 47.5%, which was lower than the EBV infection rate of 67.3% of children in a study in Beijing in 2013 and 83% in 2000 (10), although only a single antibody was selected in that study. This agrees with a decreasing yearly trend. In this study, the EBV infection rate of 10-year-old children exceeded 90% for the first time and reached the adult level, while Xiong suggested that the EBV infection rate of children in Guangzhou reached the adult level in children at the age of eight (11). We attribute this phenomenon to an increased attention to health. These differences may also be due to the methodologies used, and therefore, further research is needed to justify the validity of these findings with the same methodology. The current infection rate in this study was 15.7%. When comparing the current infection rates of each age group, we found that the preschool age group had the highest current infection rate, which was consistent with the results of most domestic studies (12, 13). This suggests that the preschool age group experienced a high incidence of EBV infection.

The fact that children who live in cities go to school at a younger age is related to an increased chance of exposure in kindergarten, which has important implications for the timing of vaccination. There was no significant difference in the EBV infection rate by sex, which was consistent with the literature (14). Li's investigation suggested that autumn and winter were the peak periods of EBV infection, which may be related to greater virus activity during these periods (15), but this study did not show this seasonal variation.

EBV infection presents a family clustering phenomenon, and 82% of siblings in families of EBV-infected children had consistent serum antibodies (16). This study shows that the EBV infection rate of children with siblings is significantly higher than that of only children. It is speculated that close contact between siblings may increase the risk of infection, but genetic factors cannot be ruled out (16). The smoking status of family members increases the rate of EBV infection in children, which is consistent with previous research (17). Additionally, providing a good living environment can reduce the risk of EBV infection. The EBV infection rate of children with dedicated tableware was significantly lower than that of children without dedicated tableware, which may be related to the main transmission route of EBV through saliva. Without their own dedicated tableware, children have a higher risk for EBV infection. This suggests the importance of strengthening the promotion of health and cultivating good hygiene.

Lifestyle is an important measure in controlling EBV infection, but parental education level is a more important independent protective factor for EBV infection in children, which is consistent with recent research (18). Due to the high prevalence of EBV infection in adults, breastfeeding increases the chance of infection in children (17). However, our study found that the EBV infection rate was lower in children breastfed for a longer time, which may indicate a correlation between EBV infection and nutritional status. This agrees with the work of Juan et al. (19), but more research is warranted.

The risk of EBV infection is higher for children in families living in rental properties (20). In this study, univariate and multivariate logistic regression models were used to analyze the data, and neither suggested this as an influencing factor of EBV infection. We speculate that renting was associated with EBV infection when more people live together in a smaller space. A smaaler space represents a higher density of people, which increases EBV infection rates. However, with the advancement of urban shantytown reform policies, rental properties may provide a better living environment and a lower density of people.

Our study had several limitations. One of the major results from the population under study is that the preschool age group had the highest acute infection rate. Indeed, we only included children who had undergone EBV-specific antibody and DNA tests; in other words, our population (hospital population) was not strictly representative of the population of children in Hangzhou. Different prevalence results might be observed if every child in Hangzhou had received EBV-related screening. Moreover, the preschool-aged children in our study were close to half of the population, and preschool-aged children are more susceptible to pathogen infection. All of the above may cause selection bias. Therefore, an unselected larger-sample clinical study is required to confirm this hypothesis. Finally, this study only contained 2 years of records (2019–2020), which showed a decreasing trend of the EBV infection rate but cannot be used to make a definitive conclusion.

## Conclusions

In summary, we found that the EBV infection rates differ by mode of detection, and the two methods should be combined to improve the rate of infection detection. This preliminary study explored the low rate of EBV infection in children at the Affiliated Hospital of Hangzhou Normal University. The infection rate was closely related to the number of siblings, family members' smoking habits, parents' education level, breastfeeding duration, and meal patterns. Factors such as sex, season, and rental housing did not play a role in infection. Specific attention should be given to current infection in the preschool age group.

## Data availability statement

The original contributions presented in the study are included in the article/supplementary material, further inquiries can be directed to the corresponding author.

## Ethics statement

The studies involving human participants were reviewed and approved by Research Ethics Committee of Hangzhou Normal University Affiliated Hospital. Written informed consent to participate in this study was provided by the participants' legal guardian/next of kin. Written informed consent was obtained from the minor(s)' legal guardian/next of kin for the publication of any potentially identifiable images or data included in this article.

## Author contributions

YY: data collection, data analysis, manuscript writing, and manuscript revision. YZ manuscript revision and project supervision. All authors read and approved the final manuscript. All authors contributed to the article and approved the submitted version.

## Funding

This study was supported by Hangzhou Science and Technology Project (20191203B107).

## Conflict of interest

The authors declare that the research was conducted in the absence of any commercial or financial relationships that could be construed as a potential conflict of interest.

## Publisher's note

All claims expressed in this article are solely those of the authors and do not necessarily represent those of their affiliated organizations, or those of the publisher, the editors and the reviewers. Any product that may be evaluated in this article, or claim that may be made by its manufacturer, is not guaranteed or endorsed by the publisher.

## Abbreviations

EBV: Epstein-Barr virus
ELISA: Enzyme-linked immunosorbent assay
EBV-CA-IgM: EBV capsid antigen IgM antibody
EBV-CA-IgG: EBV capsid antigen IgG antibody
EBV-EA-IgM: EBV early antigen IgM antibody
EBV-NA-IgG: EBV nuclear antigen IgG antibody
PCR: Polymerase chain reaction
OR: Odds rations
PBMC: peripheral blood mononuclear cell.
